# Association of interleukin-33 gene single nucleotide polymorphisms with ischemic stroke in north Chinese population

**DOI:** 10.1186/1471-2350-14-109

**Published:** 2013-10-09

**Authors:** Liang Guo, Xinghu Zhou, Xiaofan Guo, Xingang Zhang, Yingxian Sun

**Affiliations:** 1 Department of Cardiology, the First Hospital of China Medical University, Shenyang 110001, China

**Keywords:** Interleukin-33, Single nucleotide polymorphism, Ischemic stroke

## Abstract

**Background:**

IL-33, an IL-1-like cytokine, is a ligand for IL1RL1, which is an important effector molecule of type 2 T helper responses. Although IL-33/IL1RL1 interaction has been suggested to be important in the development of atherosclerosis, genetic influences of the polymorphisms of *IL33* in human ischemic stroke are unclear. The aim of this study was to examine whether the single nucleotide polymorphisms in *IL33* are associated with ischemic stroke in Northern Chinese population.

**Methods:**

We used a nested case–control study involving 90 ischemic stroke patients and 270 age-matched, sex-matched and blood pressure-matched non-ischemic stroke controls from a rural population and determined the genotypes of four polymorphisms (rs1929992, rs10975519, rs4742170, rs16924159) in *IL33* by Snapshot SNP genotyping assays to assess any links with ischemic stroke.

**Results:**

Univariate analysis showed two single nucleotide polymorphisms (rs1929992, rs4742170) in *IL33* were associated with ischemic stroke in additive, dominant, and recessive model. Binary Logistic Regression shows that rs4742170 variation is the most important factor associated with ischemic stroke (adjusted odds ratio (OR) = 1.880, 95% confidence interval (CI) = 1.316-2.686 in an additive model; OR = 2.091, CI = 1.249-3.498 in a dominant model; OR = 2.623, CI = 1.366-5.036 in a recessive model).

**Conclusion:**

In this sample of patients, genetic variation of rs4742170 in *IL33* is significantly associated with the developing of ischemic stroke.

## Background

Ischemic stroke is a common adult disability with high mortality and severe morbidity [1]. In China, the death caused by ischemic stroke account for approximately two-thirds of all death caused by strokes [2]. High blood pressure, hypercholesterolaemia and diabetes are considered as the main risk factors of ischemic stroke. Inflammation has also been demonstrated to play an important role in the development and progression of cardio-cerebral vascular disease. Local humoral and cellular immune responses modulate the inflammatory processes involved in the development of atherosclerotic lesions, as well as in the evolution of brain infarcts in stroke patients.

Although the etiology and mechanisms of ischemic stroke is not clear, it is considered that genetic components play a significant role in the pathogenesis of ischemic stroke [3]. Genetic variation is an important factor causing ischemic stroke [4]. The risk of ischemic stroke can be influenced by genetic variation in the inflammatory agents, including Interleukin-33 (IL-33), interleukin 4 (IL-4), interleukin 6 (IL-6), intercellular adhesion molecule 1 (ICA M-1), E-selectin (E-sel), chemokine (C-C motif) ligand 11 (CCL11), lymphotoxin (LTA) [1,5,6].

IL-33, also known as IL-1 family member 11, is a member of the IL-1 cytokine family. IL-33 is a ligand for Interleukin-1 receptor-like 1(IL1RL1), that is expressed mainly on activated Th2 cells and mast cells [6-8]. IL-33 is a therapeutic target as a proinflammatory mediator and has been demonstrated to play an important role in cardio-cerebral vascular disease [9,10]. In addition, IL-33 expression is decreased and consistently restricted to vascular capillaries in the brain of Alzheimer’s disease (AD) cases [11]. The genes encoding for IL-33 are both affected by common, functionally important, genetic polymorphisms, and several genetic polymorphisms have been considered to associate with AD and cedar pollinosis [11-14]. Until now, three SNPs (rs1157505, rs11792633, and rs7044343) in *IL33* gene have been reported to be associated with risk of AD in modulating cerebral amyloid angiopathy (CAA) formation in Caucasian populations. Furthermore, another study reports that rs11792633 polymorphism is still strongly associated with LOAD in Han Chinese. These polymorphisms lead to a specific decrease CAA in the brain of non-APOE ϵ4 AD cases. Although several reports suggest that polymorphisms in IL-33 gene play important roles in the development of cardio-cerebral vascular disease, the association of polymorphisms of *IL33* with human ischemic stroke is still unclear.

In this study, the *IL33* gene polymorphisms associated with history of ischemic stroke in North Chinese patients were evaluated. Four polymorphisms (rs1929992, rs10975519, rs4742170, rs16924159) in *IL33* gene were selected by Snapshot SNP genotyping assays, and then we analyzed the association between the four SNPs and the risk of ischemic stroke.

## Method

### Study population

This study was a large-scale cross-sectional epidemiological survey which was conducted in rural areas of Fuxin Country, Liaoning Province from 2004 to 2006. The methodology was described previously in detail [15,16]. A total of 6104 participants with hypertension (Blood pressure≥140/90) were recruited from 6 geographical regions in northern China. Ninety patients who suffered an ischemic stroke during a mean follow-up of 6.8 years were eligible to participate in this study. All patients had medical records of diagnosis based on brain computed tomography (CT)/MRI. Control subjects were selected in a ratio of 1:3 according to the case-control study criteria during the same period (control subjects matched for gender, age within 3 years, geographic location, and blood pressure category (<160/100, ≥160/100 and ≤180/110, >180/110 mmHg)). The research protocol was approved by China Medical University Research Ethics Committee and written informed consent was obtained from all patients or their guardians.

### Selection of SNPs and genetic analyses

*IL33* gene Polymorphisms were selected by Snapshot SNP genotypin. Haplotype block analysis was performed by Hapmap phase genotype data for the chromosomal region 9:6215786.6257983 (CHB database, Hapmap release #27). And linkage disequilibrium analysis was conducted using Haploview software (http://www.broad.mit.edu/mpg/haploview). The selection of tagSNPs was performed by running the tagger program implemented in Haploview. The criteria for *r*^2^ was set at > 0.7. It indicated that any marker that was not eventually chosen as a tagging marker was considered strongly correlated with at least one of the tagging markers with *r*^2^ > 0.7. Venous blood of patients was previously collected by venepuncture. Genomic DNA was extracted using the QIAamp DNA kit (Qiagen, Hilden, Germany). Genotyping was performed by multiplex SNaPshot technology using an ABI fluorescence-based assay discrimination method (Applied Biosystems, Foster city, CA, USA), which had been described in detail in previous studies [17,18]. Our assay was based on multiplex SNaPshot method. The *IL33* polymorphisms were identified by polymerase chain reaction (PCR) as previously described [19]. Detection of single-base extended probe primers was based on fluorescence and extended length detected by capillary electrophoresis on ABI3730XL Sequencer (Applied Biosystems, Foster City, CA, USA).

### Statistical analysis

The software SPSS for Windows, version 11.5, was used for statistical analysis. Continuous variables were expressed as mean ± SD. Data of differences between case and control groups were compared by *t* test. Categorical variables were presented as percentage. The χ^2^ or exact test was used to evaluate significant deviation from Hardy-Weinberg equilibrium. Then differences in genotype distribution of the polymorphism between case and control subjects were compared by a 2×2 contingency χ^2^ test with one degree of freedom. Logistic regression analysis was used to evaluate the association between genotype of SNPs and ischemic stroke. Three models were used for statistical analysis, including dominant model (Hz- homozygote, Het- heterozygote; for isolated SNPs: Hz(rare) + Het vs. Hz(common); for haplotypes: at least 1 of the indicated haplotypes vs. all other haplotypes), the recessive genetic model (for isolated SNPs: Hz rare vs. Het + Hz common; for haplotypes: 2 of the indicated haplotypes vs. all other haplotypes) and the additive model. The polymorphisms of *p* values were evaluated by a stepwise forward conditional logistic regression analysis in each additive model, dominant model, and recessive model, respectively. Odds ratios (ORs) were calculated with 95% CI. A *p* value of less than 0.05 was considered statistically significant.

## Results

Clinical characteristics of all the subjects in the present study were shown in Table 1. There were no significant differences between ischemic stroke group and control group in terms of mean age, sex, blood pressure class, the percentage of diabetes, hyperlipidemia, smoking, drinking, and lower education (P > 0.05). The percentage of high BP which was controlled to levels below 140/90 mm Hg was also similar between the two groups (P = 0.716).

**Table 1:** Clinical Characteristics of the Study Population

	**Case (n=90)**	**Control (n=270)**	***p*value**
Mean age, year ± SD	62.5 ± 8.5	62.1 ± 8.5	0.743
body mass index (BMI), mean ± SD	23.2 (3.0)	23.7 (4.0)	0.278
Male sex, absolute number (%)	53 (58.9)	159 (58.9)	1
Blood pressure (BP) class 3, no. (%)	30 (33.3)	89 (33)	0.948
BP controlled to below 140/90mmHg (%)	19 (21.1)	62 (23.0)	0.716
Diabetes, absolute number (%)	11 (12.2)	34 (12.6)	0.927
Hyperlipidemia, absolute number (%)	40 (44.4)	104 (38.8)	0.345
Current smoking, absolute number (%)	49 (54.4)	128 (47.4)	0.247
Drinking, absolute number (%)	33 (36.7)	96 (35.6)	0.849

**Figure 1:**
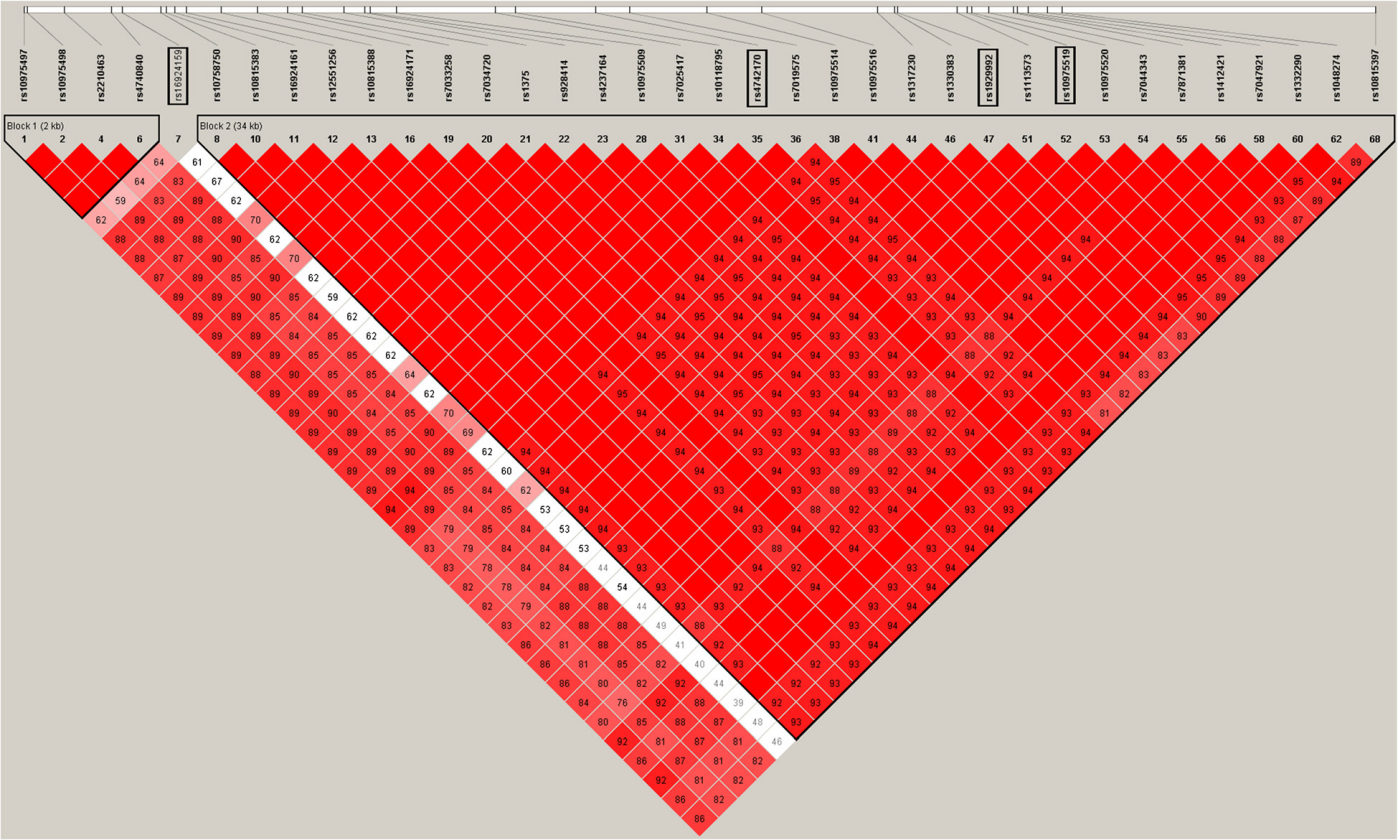
**Pairwise linkage disequilibrium between 36 SNPs as measured by r2 estimated by the Haploview 4.2 program using the HapMap CHB data set.** The boxed polymorphisms, rs4742170, rs1929992, rs10975519 and rs16924159, were genotyped in this study.

Genotype information of a total of 36 polymorphisms with a frequency >0.10 in IL33 was derived from the HapMap CHB populations (http://hapmap.ncbi.nlm.nih.gov/cgi-perl/gbrowse/hapmap27_B36/). Pairwise LD among the 36 SNPs was measured with the Haploview 4.2 program (Figure 1). We finally selected polymorphism rs4742170, rs1929992, rs10975519 and rs16924159 for association studies using tagger in the Haploview 4.2 program, and these four SNPs captured 36 of 36 alleles with a mean r2 of 0.95. Locations and Minor Allele Frequencies of the four SNPs were showed in Table 2.

**Table 2:** **Locations and minor allele frequencies (MAF) of polymorphisms in*IL33***

**Name**	**Alleles**	**MAF**	**Position**
rs16924159	G:A	0.349	5’-Flanking region
rs4742170	T:C	0.464	Intron 1
rs1929992	A:G	0.489	Intron 3
rs10975519	C:T	0.463	Exon 5

The genotype distribution of the *IL33* gene SNPs in both groups were shown in Table 3. In univariate analysis, the rs4742170 was significantly associated with ischemic stroke in the additive model, the dominant model and recessive model (additive *p*: 0.002, dominant *p*: 0.005, recessive *p*: 0.003). Furthermore, the rs1929992 also showed significantly association with ischemic stroke in the dominant model and recessive model (additive *p*: 0.025, dominant *p*: 0.054, recessive *p*: 0.014). However, the rs10975519 was associated with the risk of ischemic stroke only in the recessive model (additive *p*: 0.061, dominant *p*: 0.075, recessive *p*: 0.040), and the rs16924159 showed no association with ischemic stroke (additive *p*: 0.028, dominant *p*: 0.078, recessive *p*: 0.472). Then rs4742170 in *IL33* gene was selected for further studies.

**Table 3:** Association between SNPs and risk for development of ischemic stroke for northern Chinese population in additive, dominant, and recessive model

**Genotype**	**Case(n=90)**	**Control(n=270)**	**Additive*p***	***Dominant p***	***Recessive p***
rs1929992					
GG, absolute number (%)	28(31.1)	115(42.6)	0.025	0.054	0.014
AG, absolute number (%)	41(45.6)	121(44.8)			
AA, absolute number (%)	21(23.3)	34(12.6)			
rs10975519					
TT, absolute number (%)	25(27.8)	103(38.1)	0.061	0.075	0.040
CT, absolute number (%)	44(48.9)	129(47.8)			
CC, absolute number (%)	21(23.3)	38(14.1)			
rs4742170					
TT, absolute number (%)	26(28.9)	124(45.9)	0.002	0.005	0.003
CT, absolute number (%)	45(50.0)	121(44.8)			
CC, absolute number (%)	19(21.1)	25(9.3)			
rs16924159					
GG, absolute number (%)	52(57.8)	127(47.0)	0.208	0.078	0.472
AG, absolute number (%)	31(34.4)	115(42.6)			
AA, absolute number (%)	7(7.8)	28(10.4)			

As shown in Table 4, after adjustment for Binary Logistic Regression, the rs4742170 polymorphism was still significant in three models (adjusted odds ratio (OR) = 1.880, 95% confidence interval (CI) = 1.316-2.686, *p* value=0.001 in an additive model; OR = 2.091, 95% CI = 1.249-3.498, *p* value=0.005 in a dominant model; OR = 2.623, 95% CI = 1.366-5.036, *p* value=0.004 in a recessive model).

**Table 4:** Associated between rs4742170 variation with ischemic stroke in additive, dominant, and recessive model after adjustment for Binary Logistic Regression

	**Wald**	***p*value**	**OR**		
Additive	12.032	.001	1.880	(1.316	-2.686)
dominant	7.882	.005	2.091	(1.249	-3.498)
recessive	8.390	.004	2.623	(1.366	-5.036)

## Discussion

This study analyzed SNPs in the *IL33* gene in Chinese patients with ischemic stroke for the first time. We investigated four SNPs in *IL33* gene (rs4742170, rs1929992, rs10975519, rs16924159), and discovered two SNPs (rs4742170, rs1929992) were significantly associated with ischemic stroke at univariate analysis. Moreover, the rs4742170 polymorphism remained significant association with ischemic stroke in three models after adjustment for Binary Logistic Regression.

Many studies have reported that IL-33 could support Th2 cells, reduce macrophage foam cell formation and, probably, modulate endothelial cell function [20,21], which may play important roles in the series of events involve in the pathogenesis and development of stroke. Recently, it has been reported that IL-33/ST2 pathway can protect against atherosclerosis and adipose tissue inflammation which are well-known risk factors for ischemic stroke [21].

In this study, we reported the significant association of the rs4742170 SNP in *IL33* with ischemic stroke in Chinese population for the first time. Several studies have demonstrated the gene variation of *IL33* is associated with some cardiovascular and cerebrovascular diseases. Sakashita et al. indicate that SNP of rs1929992 in IL33 gene is significantly associated with cedar pollinosis [13]. In addition, J Chapuis et al reports IL-33 expression is decreased in Alzheimer’s disease(AD) patients, and confirm that alleles of three SNPs in *IL33*gene (rs1157505, rs11792633 and rs704343) are associated with AD in Caucasian populations; moreover, genetic variations of *IL33* are associated with a lesser degree of CAA, which contains mainly Aβ40, and the local secretion of Aβ40 may be a key determinant of AD pathology [11]. Moreover, the study of Jin-Tai Yu has shown that the minor allele of the rs11792633 polymorphism in *IL33* is significantly associated with a reduced risk of LOAD in Chinese patients [14]. These studies suggest that the characterization of IL-33 as a genetic determinant of AD indicates a potential relevant link between CAA formation, neurovascular dysfunction, alteration of immune cell functions and inflammatory process, and all of these contribute to AD. However, it is not clear how genetic variants in *IL33* might affect its function to reduce the risk of ischemic stroke.

Nevertheless, our study also had some limitations. First, it is a case-control study, all the participants in this study were Chinese people, and the possibility of ethnicity as a confounding factor could be excluded. Indeed, samples should be included subjects from different geographical and racial backgrounds which could affect the consequences of study. Second, no testing was performed to assay the functional consequences of genetic variation in individual patients, such as plasma or tissue levels of IL-33, and the association between SNPs in *IL33* and ischemic stroke is conjectural. Thirdly, the degree of atherosclerosis was not quantified in the subjects. Finally, the results of this study are based on a single-center experience and a small number of patients and must be viewed as preliminary and our results should be confirmed in larger samples and should be tested in groups of different geographical and racial backgrounds. Thus, further replication studies will be necessary to evaluate the association between the polymorphisms in *IL33* gene and the risk of ischemic stroke.

## Conclusion

In conclusion, our findings supported genetic variation of rs4742170 in *IL33* gene as a modest protective factor for ischemic stroke in North Chinese, and provided further contribution toward new opportunities to investigate ischemic stroke pathogenesis, treatment, and prevention.

## Competing interests

The authors declare that they have no competing interests.

## Authors’ contributions

Dr SY, GL and ZX, have all participated in the conception and design of the study and all authors (Dr SY, GL, ZX, GX and ZX) have contributed in the analysis and interpretation of data and also in the drafting and revising of the manuscript. Before submission each author have read and the given final approval of the manuscript. The manuscript has not been published and is not being considered for publication in whole or part in any language. All authors read and approved the final manuscript.

## Pre-publication history

The pre-publication history for this paper can be accessed here:

http://www.biomedcentral.com/1471-2350/14/109/prepub
